# Dietary Habits and Relationship with the Presence of Main and Trace Elements, Bisphenol A, Tetrabromobisphenol A, and the Lipid, Microbiological and Immunological Profiles of Breast Milk

**DOI:** 10.3390/nu13124346

**Published:** 2021-12-02

**Authors:** Irma Castro, Rebeca Arroyo, Marina Aparicio, María Ángeles Martínez, Joaquim Rovira, Susana Ares, Sara Cristina Cunha, Susana Casal, Jose Oliveira Fernandes, Marta Schuhmacher, Martí Nadal, Juan Miguel Rodríguez, Leónides Fernández

**Affiliations:** 1 Department of Nutrition and Food Science, Complutense University of Madrid, 28040 Madrid, Spain; irmacastro@ucm.es (I.C.); rebecaa@ucm.es (R.A.); marinaap@ucm.es (M.A.); jmrodrig@ucm.es (J.M.R.); 2 Centro de Investigación Biomédica en Red Fisiopatología de la Obesidad y la Nutrición (CIBEROBN), Institute of Health Carlos III, 28029 Madrid, Spain; mangeles.martinez@urv.cat; 3 Unitat de Nutrició, Departament de Bioquímica i Biotecnologia, Universitat Rovira i Virgili, 43201 Reus, Spain; 4 Nutrition Unit, University Hospital of Sant Joan de Reus, 43204 Reus, Spain; 5 Institut d’Investigació Sanitària Pere Virgili (IISPV), 43007 Reus, Spain; 6 Environmental Engineering Laboratory, Departament d’Enginyeria Quimica, Universitat Rovira i Virgili, Av. Països Catalans 26, 43007 Tarragona, Spain; joaquim.rovira@urv.cat (J.R.); marta.schuhmacher@urv.cat (M.S.); 7 Laboratory of Toxicology and Environmental Health, School of Medicine, IISPV, Universitat Rovira i Virgili, Sant Llorenç 21, 43201 Reus, Spain; marti.nadal@urv.cat; 8 Department of Neonatology, Universitary Hospital La Paz, P° de la Castellana, 261, 28046 Madrid, Spain; susana.ares@salud.madrid.org; 9 LAQV-REQUIMTE, Laboratory of Bromatology and Hydrology, Faculty of Pharmacy, University of Porto, 4050-313 Porto, Portugal; sara.cunha@ff.up.pt (S.C.C.); sucasal@ff.up.pt (S.C.); josefer@ff.up.pt (J.O.F.); 10 Department of Galenic Pharmacy and Food Technology, Complutense University of Madrid, 28040 Madrid, Spain

**Keywords:** human milk, milk microbiota, immunology, bisphenol A, chemical elements, breastfeeding, diet

## Abstract

Breastfeeding is the best way to feed an infant, although it can also be a source of abiotic contaminants such as heavy metals or bisphenol A (BPA). The early life exposure to these compounds can lead to serious toxic effects in both the short and long-term. These substances can reach breast milk through the mother’s habits, diet being one of the main routes of exposure. The aim of the present work was to analyse possible associations between the dietary habits of women and the content of major trace elements, BPA, fatty acids and lipids, and the microbiological and immunological profiles of human milk. Possible associations between major trace elements and BPA and the lipid, microbiological and immunological profiles were also analysed. The results of this study support that the microbiological composition of human milk is associated with the dietary habits of the women, and that the consumption of canned drinks is related to the presence of BPA in human milk. Furthermore, some relationships were found between the amount of major trace elements and the microbiological and immunological profile of the milk samples. Finally, the presence of BPA was associated with changes in the immunological profile of human milk.

## 1. Introduction

Breastfeeding is the ideal infant feeding option [1,2,3]. However, it can act as a source of abiotic contaminants, such as heavy metals or bisphenol A (BPA) [4,5,6,7]. Chemical substances may reach milk from the mother’s diet, the use of medication and/or drug consumption, chemical inhalation or dermal exposure. The risk associated with these contaminants increases if they bioaccumulate in the adipose tissue (including mammary tissue) throughout a women’s life and are mobilized during pregnancy and lactation [6,8]. The toxic properties of some metallic and metalloid elements, such as mercury (Hg), lead (Pb) or cadmium (Cd), include neurotoxic effects, preterm birth or intrauterine growth retardation. Furthermore, they can also act as mutagenic and carcinogenic agents or endocrine disruptors [9,10]. Tetrabromobisphenol A (TBBPA) is another chemical of concern, which has a widespread use as flame retardant. This persistent environmental chemical accumulates in the body and the human milk acts as a vehicle from the mother to the infant [11,12]. The exposure to TBBPA during early infant development may contribute to the increasing incidence of disorders such as infertility, genital tract abnormalities, obesity, attention deficit hyperactivity disorder, and prostate and breast cancer later in life [13,14,15]. 

The composition of human milk varies within a feeding, over lactation and it is influenced by maternal diet, health status and environmental factors [16]. In addition, the different milk components may establish interactions between each other. The cell wall of some Gram-positive bacteria binds metals and toxic compounds, as has been observed in environmental lactic acid bacteria [17]. Accordingly, human microbiota, and in particular milk microbiota, could influence the mother-child transfer of chemical substances. The interactions between BPA and gut microbiota [18,19], and changes in the immune profile related to the presence of this undesirable substance have also been reported [20]. Therefore, knowledge of the complex composition of human milk and the interactions between its different components is relevant to gaining better understanding of all the factors modulating this complex biofluid and, hence, its impact on infant nutrition and growth.

In a previous study, the main and trace elements, BPA, TBBPA and fatty acids (FAs) contents in human milk samples were analysed and compared with those present in infant formulas [21]. The objective of the present work was to analyse potential relationships between women’s dietary habits and the microbiological and immunological profile and the content of main and trace elements, FAs, BPA and TBBPA in human milk. In addition, the correlations that may exist between all these milk components were explored.

## 2. Materials and Methods

### 2.1. Demographic Characteristics 

A total of 53 lactating women were recruited for this study through the Neonatology Service of La Paz University Hospital (Madrid, Spain). To be enrolled as a participant in the study women had to report having a healthy breastfed infant, born at term after an uncomplicated pregnancy, and having healthy nursing without any symptoms related to breast infection or breast pain from birth to recruitment. Volunteers provided specific information on age, weight, height and lactation time when the milk sample was collected.

The mean (95% CI) age of the participants (*n* = 53) was 35 (34,36) years, ranging from 25 to 43 years (Table 1). The median (IQR) body mass index (BMI) was 24.5 kg/m^2^ (20.89–27.19 kg/m^2^). Most women provided the milk sample during the first six months of lactation, while 19% of the samples were collected after the sixth month of lactation. All volunteers gave written informed consent to the protocol (C.P.-C.I. 10/017-E), which was previously approved by the Ethical Committee of Clinical Research of La Paz University Hospital (Madrid, Spain). Some women did not provide some of the demographic and lactation data requested in the questionnaires (Table 1).

### 2.2. Food Frequency Questionnaires

The participants completed a Food Frequency Questionnaire (FFQ; Supplementary Material) to evaluate their dietary habits, which was specifically designed for this study by the Center of Environmental, Food and Toxicological Technology (TecnATox, Rovira i Virgili University, Reus, Spain). The questionnaire included information about the frequency of intake and the consumption level of food items in a finite list. Most of the food items belonged to the following three main groups: fish (fresh tuna, canned tuna, salmon, swordfish, other fish), cereals (bread, pasta, rice), and dairy products (milk, cheese, yogurt). In addition, the questionnaire inquired about the ingestion of some specific products, such as nuts, tap water, bottled water, canned drinks and microwavable food. Servings from the same group of food were added together and the results were transformed to the same unit of measure (servings per week). 

### 2.3. Milk Sampling

Milk samples were collected aseptically from each woman by manual expression after cleansing of the breast with soap and water. The samples were collected in tubes of different materials depending on the posterior analysis: plastic sterile tubes for microbiological and immunological analysis, and glass tubes for metals, metalloids, Fas, BPA, and TBBPA analysis. Milk was cooled immediately and kept at 4 °C in cool boxes until arrival at the laboratory where samples were divided into aliquots and frozen (−20 °C). In order to eliminate or minimize potential lab biases, all the samples were submitted only to a single freeze-thaw cycle and were analysed by the same researchers using the same batch of reagents and equipment. 

### 2.4. Bacteriological Analyses of Milk Samples

Milk samples were plated onto Columbia Nalidixic Acid (CNA; a medium particularly suitable for isolation of streptococci, staphylococci, enterococci, corynebacteria and related Gram-positive bacteria; BioMérieux, Marcy-l’Etoile, France), MacConkey agar (MCK; a medium for the isolation of Gram-negative bacteria; BioMérieux), De Man, Rogosa and Sharpe supplemented with L-cysteine (0.05%, w/v) agar plates, MRS-Cys; a medium for the isolation of lactic acid bacteria (Oxoid, Basingstoke, UK), and Wilkins-Chalgren agar (WC; a medium for isolation of strict anaerobe bacteria; Oxoid, UK). MCK and CNA plates were incubated under aerobiosis at 37 °C for 24 and 48 h, respectively. MRS-Cys and WC plates were incubated anaerobically (85% nitrogen, 10% hydrogen, 5% carbon dioxide) in an anaerobic workstation (MINI-MACS, DW Scientific, Shipley, UK) at 37 °C for 48 h. After incubation, bacterial counts in each medium were recorded and, subsequently, at least one representative of each colony morphology was selected from the agar plates. The isolates were identified by Matrix Assisted Laser Desorption Ionization-Time of Flight (MALDI-TOF) mass spectrometry using a Vitek-MS instrument (BioMérieux), following the protocol described by Martín et al. [22].

### 2.5. DNA Extraction from Milk Samples and PCR Amplification and Sequencing

DNA extraction was done following the protocol described by Lackey et al. [23] for milk samples. Total eluted DNA was quantified using a NanoDrop ND-1000 UV spectrophotometer (Nano-Drop Technologies, Wilmington, DE, USA).

A dual-barcoded 2-step PCR reaction was conducted to amplify a fragment of the V3–V4 hypervariable region of the bacterial 16S ribosomal RNA (rRNA) gene. Equimolar concentrations of the universal primers S-D-Bact-0341-b-S-17 (ACACTGACGACATGGTTCTACACCTACGGGNGGCWGCAG) and S-D-Bact-0785-a-A-21 (TACGGTAGCAGAGACTTGGTCTGACTACHVGGGTATCTAATCC) were used as previously described [24] with the following modifications. Twenty-six PCR cycles were performed with an annealing temperature adjusted to 50°C, generating amplicons of approximately 464 bp from the V3–V4 hypervariable region. The primers were synthesized by Isogen Life Sciences (Castelldefels, Spain). Barcodes used for Illumina sequencing were appended to 3′ and 5′ terminal ends of the PCR amplicons to allow for the separation of forward and reverse sequences. 

An automated electrophoresis system (2100 Bioanalyzer, Agilent Technologies, Palo Alto, CA, USA) was used to determine the DNA concentration in each sample. Barcoded PCR products from all samples were pooled at approximately equimolar DNA concentrations and run on a preparative agarose gel. The correct sized band was excised and purified using the QIAEX II Gel Extraction Kit (Qiagen, Hilden, Germany) and then quantified using PicoGreen (BMG Labtech, Jena, Germany). Finally, one aliquot of pooled, purified, barcoded DNA amplicons was sequenced using the Illumina MiSeq pair-end protocol (Illumina Inc., San Diego, CA, USA) at the facilities of the Scientific Park of Madrid (Spain). The resulting sequences are available in the BioSample database of the National Center for Biotechnology Information (Bethesda, MD, USA) under the BioProject ID PRJNA715929.

The amplified fragments were taxonomically analyzed using the Illumina™ software version 2.6.2.3, according to the manufacturer’s guidelines and pipelines. The resulting high-quality reads were assembled and classified taxonomically into operational taxonomic units (OTUs) by comparison with the Illumina-curated version of the GreenGenes taxonomic database (Illumina software version 2.6.2.3; https://www.illumina.com accessed on 7 March 2017) using a Bayesian classification method and a level of similarity of at least 97%. Bacterial taxa abundances were normalized to the total number of sequences in each sample and expressed as relative abundances. Negative controls were processed in parallel with samples starting at DNA extraction, and no amplification was observed. In order to filter out contaminant sequences, the “isContaminant” function in the “decontam” package [25] in R was used [26].

### 2.6. Immunological Analysis of Milk Samples

The concentrations of 23 immune factors, including innate immune factors (IL1β, IL6, IL12, IFNγ, TNFα), acquired immunity factors (IL2, IL4, IL10, IL13, IL17), immunoglobulins (Igs) (IgA, total IgG [IgGt], IgM), chemokines (IL8, Groα, MCP1, MIP1β), and growth factors (IL5, IL7, granulocyte colony-stimulating factor (GCSF), granulocyte–macrophage colony-stimulating factor (GMCSF), TGFβ_2_, and epidermal growth factor [EGF]) were determined by magnetic bead-based multiplex immunoassays using a Bioplex 200 instrument (Bio-Rad, Hercules, California, USA) and the Bio-Plex Pro Human Cytokine, Chemokine, and Growth Factor Assays (Bio-Rad), according to manufacturer’s instructions. EGF was determined by ELISA using the RayBio Human EGF ELISA kit (RayBiotech, Norcross, GA, USA). Concentrations of Igs (IgA, IgGt, and IgM) were determined using the Bio-Plex Pro Human Isotyping Assay kit (Bio-Rad) in the Bioplex system instrument.

Prior to their analysis, milk samples (1 mL) were centrifuged (800× *g*, 15 min, 4 °C), and after removing the upper fat layer, the supernatant was transferred and aliquoted into different tubes for subsequent immunological analysis. A fresh aliquot was used for each assay, avoiding defrosting cycles. Every assay was run in duplicate according to the manufacturer’s instructions, and standard curves were performed for each analyte in every assay. The inter-assay coefficients of variation were below manufacturers’ instructions for all the immune markers. Concentrations of immunological compounds were expressed as the weight per volume of whole milk.

### 2.7. Main and Trace Elements, BPA, TBBPA and FAs Analysis of Milk Samples

The analysis of main and trace elements, BPA, TBBPA, and FAs was done and previously published by Martínez et al. [21]. Only main and trace elements detected in at least 25% of the samples were included in this work: chromium (Cr), copper (Cu), calcium (Ca), potassium (K), sodium (Na), strontium (Sr), selenium (Se), magnesium (Mg), zinc (Zn), barium (Ba) and Cd. Regarding the lipid profile, only relevant lipid classes and FAs (according to their frequency of detection and concentration and/or bioactive characteristics), including total fat, saturated FAs (SFA), monounsaturated FAs (MUFA), ω-3, ω-6 and total polyunsaturated FAs (PUFA), trans FAs (TFA), lauric acid (12:0), myristic acid (14:0), palmitoleic acid (16:1), stearic acid (18:0), oleic acid (18:1), linoleic acid (18:2), conjugated linoleic acid (CLA; 18:2, *c*9,*t*11), α-linolenic acid (18:3 n-3), arachidonic acid (ARA; 20:4 n-6), eicosapentaenoic acid (EPA; 20:5 n-3) and docosahexaenoic acid (DHA; 22:6 n-3), were taken into account in this work. 

In some cases, the sample volume was insufficient to perform all the planned analyses, so the priority order for analysis was the following: immunological and culture-independent microbiological analysis, main and trace elements, BPA, TBBPA, FAs and culture-dependent microbiological analysis. The number of samples included in each analysis is specified in each table and figure.

### 2.8. Statistical Analysis

Distribution of the data was evaluated using the Shapiro-Wilk normality test. Continuous variables conforming to a normal distribution were expressed as the mean and the 95% confidence interval (95% CI), while those that did not follow a normal distribution were expressed as the median and the interquartile range (IQR). 

Main and trace elements, BPA, TBBPA and FAs contents were expressed per volume of milk. Microbiological data, recorded as colony-forming units (CFU) per mL of milk, immunological compounds and main and trace elements concentrations were transformed to logarithmic values before statistical analysis. 

In order to discard confounding factors, generalized linear models (GLM) and the Kruskal-Wallis test were performed to ascertain the influence of age, body mass index and time of lactation on all parameters analysed. 

A Principal Component Analysis (PCA) was performed to identify patterns for the consumption of the main food groups among the participants using the “FactoMineR” package [27] in R [26]. A method to reduce variables (*cos*^2^ > 0.2) was used to identify the most important parameters in the distribution of the samples. Hierarchical clustering on principal components using Ward’s method (agglomerative hierarchical clustering procedure) was performed to group the participants by dietary similarities. Biplots of individuals and variables were drawn with the “factoextra” R package [28]. In order to identify differences in the concentration and the frequency of detection of the variables (microbiological counts, relative abundance of bacterial taxa, and the content of immunological compounds, FAs, main and trace elements, BPA and TBBPA) between groups with different dietary habits, Kruskal-Wallis tests with Bonferroni adjustment for multiple comparisons and Fisher tests were used. 

Correlation between the log transformed bacterial counts and the relative abundance of OTUs in milk samples for selected bacterial genera were established using Pearson correlation analysis. The strength and direction of association between variables (main and trace elements, free and total BPA and TBBPA content with culture-dependent and culture-independent results, and the concentration of immunological compounds) was observed using a scatter plot of the data and quantified after performing Spearman’s rank-order correlation analyses. The correlation matrix was visualized using the function “corrplot” in the R package [29].

Main and trace elements contents were stratified according to the median value, and BPA was categorized according to its presence or absence. Differences between the resulting groups of samples were analyzed using parametric ANOVA tests or nonparametric Kruskal-Wallis tests, depending on the distribution of the variable. 

Significance for all statistical tests was declared at *p* ≤ 0.05. All statistical analysis were performed with the software R statistic, version 3.6.0 (R-project, http://www.r-project.org accessed on 29 October 2021).

## 3. Results

### 3.1. Microbiological, Immunological, Lipid, Main and Trace Elements, BPA, and TBBPA Contents in Milk Samples

The results of the microbial, immunological, lipid, main and trace elements, BPA and TBBPA analyses are presented in Supplementary Tables S1–S4. They are briefly summarized below.

#### 3.1.1. Microbial Profile of Milk Samples

Microbiological characterization of milk samples using culture-dependent methods indicated that bacterial growth was observed in 47 of the 48 samples analysed (Supplementary Table S1). The mean total bacterial count in the milk samples with detectable growth was 4.77 log_10_ CFU/mL, with values ranging from 1.30 to 5.93 log_10_ CFU/mL. *Staphylococcus epidermidis* was the bacterial species most frequently isolated from the samples (90%), and it was also the most abundant with mean (95% CI) counts of 4.73 log_10_ UFC/mL (3.98, 4.99 log_10_ UFC/mL) (Supplementary Table S1). *Staphylococcus aureus* and *Staphylococcus lugdunensis* were detected in 25% and 15% of the samples, respectively, but at lower abundance than *S. epidermidis*. In addition, *Enterococcus faecalis* and *Enterobacteriaceae* isolates were present in at least 10% of the samples. The rest of the bacterial species or genera identified were detected in less than 10% of the samples (Supplementary Table S1). Most of the isolates (from CNA and WC plates) were Gram-positive, being identified as members of the genera *Staphylococcus, Streptococcus* and *Corynebacterium*. *Lactobacillus* and *Bifidobacterium* isolates were found at a lower frequency and only in MRS-Cys and/or WC plates incubated in anaerobic conditions. A few Gram-negative isolates were recognized from MCK plates, and all were identified as *Enterobacter* and *Klebsiella* strains.

Microbiological characterization of milk samples was also performed using culture-independent methods (Supplementary Table S2). A total of 50 human milk samples were sequenced targeting the V3–V4 rRNA hypervariable region resulting in 6,694,925 usable reads (mean ± SD = 133,898 ± 15,765 reads/sample, ranging from 178,598 to 107,925 reads/sample) and 2,686 OTUs (median (IQR) = 700 OTUs/sample (609–797) OTUs/sample). The great majority of the OTUs were classified into the domain Bacteria (median [IQR] = 100% [99.994–99.998%]), but a few OTUs corresponded to domain Archaea (median [IQR] = 0.003% [0.002–0.006%]) in 47 samples. Assembled OTUs were assigned to known 27 phyla, 60 classes, 116 orders, 257 families, and 698 genera.

At the phylum level, Firmicutes, Proteobacteria, and Actinobacteria were detected in all the samples analyzed with a relative abundance greater than 1% (Supplementary Table S2). The most abundant phylum was Firmicutes, which was present in most of the samples (*n* = 39) at the highest relative abundance (77.90%). In the rest of the samples (*n* = 11), Actinobacteria was the phylum with the highest relative abundance (6.76%). Minor phyla included all the OTUs that were not present in all the samples, or their relative abundance were less than 0.01%. Unclassified OTUs were detected also in all the samples with a median relative abundance of 0.43% at the phylum level and 5.01% at the genus level. At the genus level, *Staphylococcus,* with a median (IQR) relative abundance of 30.20% (11.08–63.59%), and *Streptococcus* (7.78% (2.14–31.97%) comprised the largest proportion of sequences in milk samples (Supplementary Table S2). There was a positive correlation between the log transformed staphylococcal counts and the relative abundance of staphylococcal OTUs (Pearson’s correlation; *r*(38) = 0.58, *p* < 0.001). Other genera showed median relative abundances less than 1%: *Rothia*, *Corynebacterium* and *Pseudomonas* showed median relative abundances greater than 0.50%; *Bacillus*, *Lactobacillus*, *Paenibacillus* and *Propionibacterium* greater than 0.10%; and *Macrococcus*, *Enterococcus*, *Clostridium*, *Kocuria*, *Bifidobacterium* and *Acinetobacter* less than 0.10%.

#### 3.1.2. Immunological Profile of Milk Samples

The immunological profile of the milk samples, including both the prevalence and concentration of 19 immunological compounds, is shown in Supplementary Table S3. Only four compounds (IL12, IL10, IL5 and GMCSF) out of the 23 analyzed were not detected in any sample. The frequency of detection and the concentration of the different immunological factors were highly variable (Supplementary Table S3). IgA, IgGt, IgM, IL8, Groα, EGF and TGFβ_2_ were found in all samples. The immunoglobulins (Ig) were the compounds found in higher concentration, especially IgA (median (IQR) = 2796.8 mg/L [1773.5–4057.2 mg/L]). High levels were observed also for EGF (5.65 µg/L (4.67–6.91 µg/L)), followed by Groα and TGFβ_2_ (2.97 µg/L (0.30–5.81 µg/L) and 1.74 µg/L (0.60–4.02 µg/L), respectively). IL1β, TNFα, IL7 and MIP1β were detected in most of the samples (78–88%), IL7 concentration (96.22 ng/L (37.10–150.68 ng/L)) being remarkably higher among these compounds. MCP1 and GCSF were found in about half of the samples, although at very different concentrations. Finally, IL6, IFNγ, IL2, IL4, IL13 and IL17 were detected in less than 30% of the samples, and their concentrations were lower than 25 ng/L. 

#### 3.1.3. Lipid, Main and Trace Elements, BPA and TBBPA in Milk Samples

The characterization of the milk samples with regard to their main and trace elements, FAs profile, and BPA and TBBPA content is provided in Supplementary Table S4.

Free and total BPA were detected in 18 and 49% of the milk samples, respectively, with a median (IQR) concentration of 0.61 µg/L (0.18–1.00) µg/L and 0.54 µg/L (0.32–1.25) µg/L, respectively. TBBPA was detected only in only three samples, with a mean concentration of 2.20 (1.60–2.25) µg/L (Supplementary Table S4).

Regarding the content of main elements, traces of K, Ca and Na were found in all the samples. Their median (IQR) values were 500.79 mg/L (439.37–580.89 mg/L), 260.81 mg/L (226.52–300.35 mg/L) and 125.22 mg/L (97.48 -177.31 mg/L), respectively. Mg was detected in 75% of the samples with a median (IQR) concentration of 33.35 mg/L (31.23–37.88) mg/L. In relation to trace elements, Cu and Cr were identified in all the samples although their concentrations never exceeded 0.5 mg/L. In contrast, the percentage of samples where Zn was detected was lower (55%), although its concentration was about four-fold higher (2.09 mg/L (0.95–4.18) mg/L). Ba, Cd, Sr and Se had the lowest concentrations (< 0.10 mg/L) and, while Se and Sr were present in most of the samples (>82%), Ba and Cd were detected only in 18 and 14% of them, respectively (Supplementary Table S4). 

Finally, the median (IQR) percentage of fat in the samples was 3.70% (2.80–4.46%) (Supplementary Table S4). All the individual and grouped FAs analysed were found in all samples. The major FA was oleic acid with a median (IQR) concentration of 1.36 g/L (1.02–1.57) g/L, followed by linoleic acid (0.43 g/L [0.32–0.60] g/L). Some essential FAs, such as α-linolenic acid, ARA and DHA, were detected at lower concentration (18.63 mg/L (14.66–25.93) mg/L, 16.14 mg/L (12.12–20.69) mg/L and 13.15 mg/L (7.93–17.97) mg/L, respectively). Globally, MUFA and SFA were the major grouped FAs found in the samples (1.45 g/L (1.12–1.68) g/L and 1.39 g/L (1.05–1.83) g/L, respectively).

A preliminary analysis examined the contribution of participant’s age, BMI and time of lactation on the microbiological, immunological and lipid profile and on the main and trace elements, BPA, and TBBPA contents in this set of milk samples. The relative abundance of *Streptococcus* was higher (Kruskal-Wallis test; *p =* 0.008) in the group of samples from women aged 35 years or younger (*n* = 22) (median (IQR) = 27.75% (4.97–45.10%)) compared to the group of women aged > 35 years (*n* = 23) (5.22% (1.41–17.28%)). The concentration of Cu was higher in the milk samples provided during the first month of lactation (*n* = 16) (median (IQR) = 0.44 mg/L [0.29–0.54] mg/L) than in samples provided from 1 to 6 month of lactation (*n* = 16) (0.26 mg/L [0.15–0.33] mg/L) (Bonferroni-adjusted Kruskal-Wallis test; *p =* 0.013). No significant relationships between the BMI and the analysed milk parameters were found. 

### 3.2. Dietary Habits of the Participant Women

A total of 49 out of the 53 participants completed the FFQ and the aggregated results are shown in Table 2. Dairy products were most frequently consumed by the participants (median (IQR) consumption of 23 servings/week (16–31 servings/week)) followed by the cereals group (16 servings/week (9–18 servings/week)). The ingestion of fish and nuts was considerably lower (1.50 servings/week (1.00–2.57 servings/week) and 1.00 servings/week (0.40–3.00 servings/week), respectively).

The intake of microwavable food was infrequent; only 15 (36%) participants ever consumed this kind of food, and only five of them did at least once a week. Regarding drinks, tap water was the most consumed beverage (42 servings/week (28–56 servings/week)), followed by canned drinks (2.00 servings/week (1.00–4.25 servings/week)) and bottled water (1.50 servings/week (0.00–19.00 servings/week)). 

**Figure 1:**
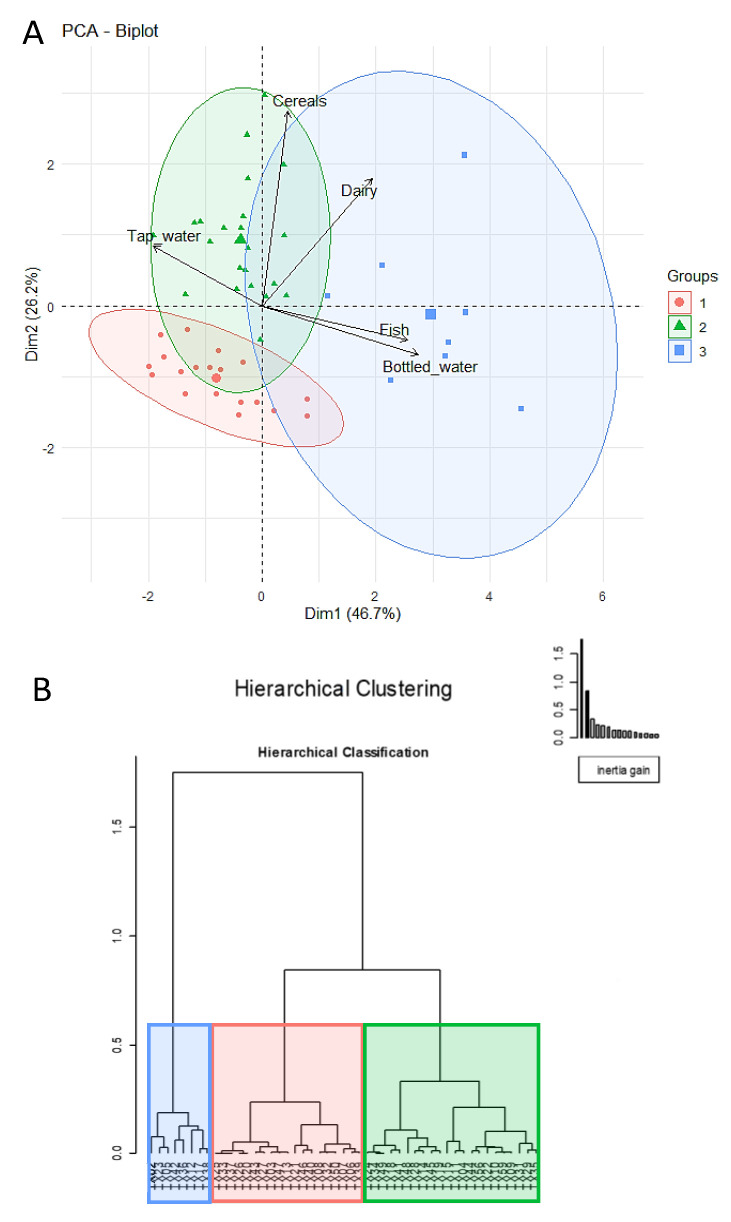
(**A**) Principal Component Analysis (PCA) of the consumption of each food group according to the dietary habits of the women participating in the study. The variables with a *cos*^2^ > 0.2 are represented as black arrows. Samples in Cluster 1 are represented as red dots, in Cluster 2 as green triangles, and in Cluster 3 as blue squares. Ellipses were obtained at a significance of 95%. The dairy products group includes milk, cheese and yogurt; the cereals group includes bread, pasta and rice, and the fish group includes fresh tuna, canned tuna, salmon, swordfish and other fish. (**B**) Dendrogram generated by hierarchical clustering on principal components using Ward’s method (agglomerative hierarchical clustering procedure) of the women participating in the study according to their dietary habits.

To ascertain if there were specific dietary patterns from the information gathered about food consumption, an exploratory PCA was performed revealing three dietary patterns according to the intake of dairy products, cereals, fish, nuts, microwavable food, canned drinks and bottle and tap water (Figure 1A).

The first two principal components (or two linear combinations of the original variables) had an *eigen* value > 1 and explained 72% of the observed variance in the women’s dietary habits. The intake of fish, dairy products, bottled and tap water had a great contribution (>10) to the first principal component (Dimension 1) that explained 46.7% of the variability, while the consumption of cereals and dairy products were the most influential (contribution > 10) in the second principal component (Dimension 2) and explained 26.2% of the total variance.

**Figure 2:**
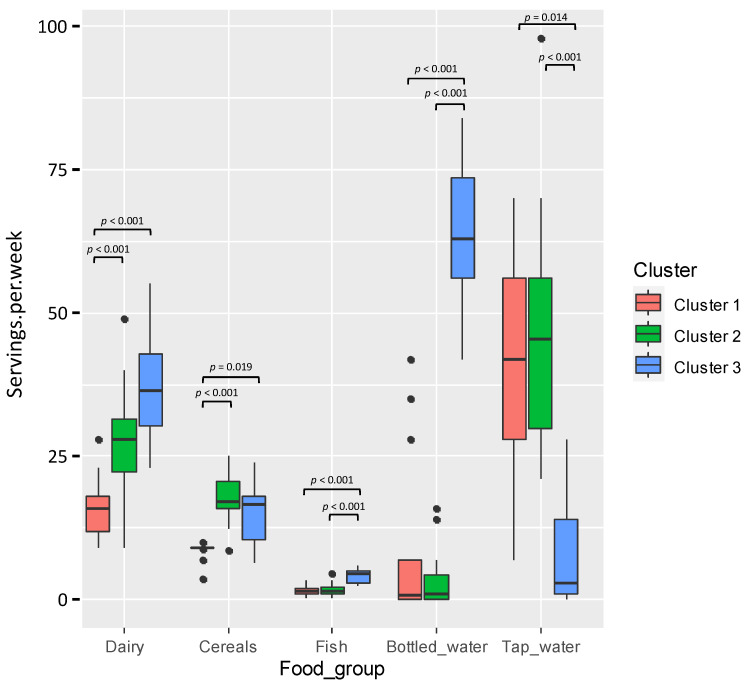
Box-plots showing the consumption of each food group (servings/week) according to the dietary habits of the women participating in the study. In the box-plots, the box represents the values of the interquartile ranges, and the median is represented as a line in the box. Outliers are represented as dots. Cluster 1 (*n* = 19) is coloured in red, Cluster 2 (*n* = 22) in green and Cluster 3 (*n* = 8) in blue. The dairy products group includes milk, cheese and yogurt; the cereals group includes bread, pasta and rice, and the fish group includes fresh tuna, canned tuna, salmon, swordfish and other fish.

The hierarchical clustering according to the dietary habits grouped women in three groups (nesting distance of 0.85), as represented in the biplot (Figure 1A,B). The differences in the dietary habits of the participants are illustrated in Figure 2. The diets of women grouped in the cluster 1 were characterized by a limited consumption of fish, dairy products and cereals, while the women in cluster 2 had higher consumption of cereals and tap water, and a very low intake of bottled water. The diets of women grouped in cluster 3 were distinguished by high consumption of fish, dairy products and bottled water, but a scarce intake of tap water. No differences in age and lactation period were observed between the groups based on dietary habits (Bonferroni-adjusted Kruskal-Wallis tests; *p* > 0.308). However, differences in BMI were observed between women grouped in cluster 2 (median (IQR) = 22.18 kg/m^2^ (20.60–24.48 kg/m^2^) and cluster 3 (median (IQR) = 26.67 kg/m^2^ (25.99–31.83 kg/m^2^) (Bonferroni adjusted Kruskal-Wallis test; *p* = 0.018).

#### 3.2.1. Variation of the Milk Bacterial Profile with the Women’s Dietary Habits

**Figure 3:**
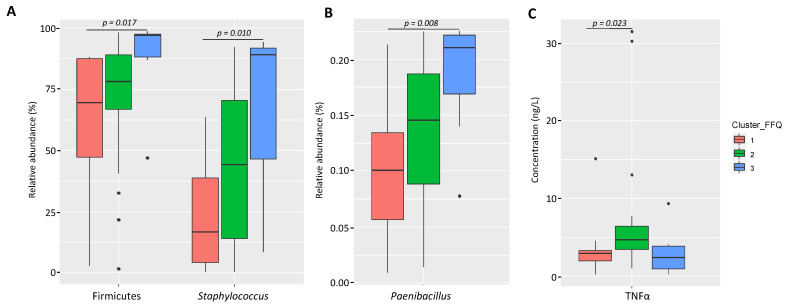
Box-plots showing the relative abundances of Firmicutes, *Staphylococcus* and *Paenibacillus* (**A**,**B**) and the concentration of TNFα (**C**) according to the dietary habits of women participating in the study. In the box-plots, the box represents the values of the interquartile ranges, and the median is represented as a line in the box. Outliers are represented as dots. Cluster 1 (*n* = 19) is coloured in red, Cluster 2 (*n* = 22) in green and Cluster 3 (*n* = 8) in blue.

No statistically significant differences were observed either in the prevalence or in the concentrations of the bacterial isolates found in milk samples using culture-dependent microbiological methods according to the women’s dietary habits (Supplementary Table S5). However, the culture-independent analysis of the microbial community in milk samples indicated that the relative abundance of Firmicutes was higher in samples from participants grouped in cluster 3 (median (IQR) = 97.09% (88.14–97.41%)) compared to samples from participants in Cluster 1 (69.61% [47.29–87.57%]) (Bonferroni-adjusted Kruskal-Wallis test; *p =* 0.017) (Figure 3A and Supplementary Table S6). This difference was also observed at the genus level since the median [IQR] relative abundances of the genera *Staphylococcus* and *Paenibacillus* in cluster 3 (89.16% (46.61–91.87%) and 0.21% (0.17–0.22%)) were statistically higher than in Cluster 1 (16.59% (4.17–38.68%) and 0.10% (0.06%–0.13%) (Bonferroni-adjusted Kruskal-Wallis tests; *p* = 0.030 and *p* = 0.013, respectively) (Figure 3A,B and Supplementary Table S6). 

#### 3.2.2. Variation of the Milk Immunological Profile with the Women’s Dietary Habits

A comparison of the prevalence and concentration of the different immunological compounds in milk samples from women with different dietary habits is shown in Figure 3C and Supplementary Table S7. The concentration of TNFα was higher in Cluster 2 (median (IQR) = 4.72 ng/L (3.43–6.42) ng/L) than in Cluster 1 (median (IQR) = 2.94 ng/L (1.96–3.33) ng/L) (Bonferroni-adjusted Kruskal-Wallis test; *p =* 0.023). No other statistical differences were observed regarding the prevalence or concentration of any other immunological compound according to the three dietary clusters (Supplementary Table S7). 

#### 3.2.3. Variation of the Milk Main and Trace Elements Content, Fatty Acids Profile, and BPA and TBBPA Content in Milk with the Women’s Dietary Habits

The profile of main and trace elements (Ba, Ca, Cd, Cr, Cu, K, Na, Se, Sr, and Zn) was similar in milk samples from the three dietary clusters, except for Mg (Supplementary Table S8). The prevalence of this element was lower in cluster 3 (detected in 25% of the samples) than in Cluster 1 and 2 (detected in 89% and 82% of the samples, respectively) (Fisher exact tests; *p =* 0.020 and *p =* 0.007, respectively). However, there were no differences in the level of Mg in the samples where it was detected between the three clusters (Supplementary Table S8). Similarly, no differences were found in the lipid profile (prevalence or concentration of the most relevant FAs, either individually or grouped, included in this study) in milk samples based on their inclusion in the three dietary clusters (Supplementary Table S9). In contrast, remarkable differences were noted in relation to the concentration of free and total BPA (Table 3). First, free BPA was found in 26% and 50% of the milk samples from women in Clusters 1 and 3, respectively, but it was not detected in any of the samples from women in Cluster 2 (χ^2^ test; *p* < 0.010). Second, the analysis of total BPA revealed that this compound was present in about half of the samples from each of the three clusters, but the median (IQR) concentration was lower in samples from women grouped in Cluster 2 (0.32 µg/L (0.20–0.36] µg/L)) than in the samples from Cluster 1 and 3 (1.03 µg/L (0.60–1.63) µg/L and 0.45 µg/L (0.34–4.53) µg/L, respectively) (Bonferroni-adjusted Kruskal-Wallis test; *p* < 0.048) (Table 3). Therefore, the relationships between consumption of individual food groups and the concentrations of free BPA, total BPA and TBBPA in milk samples were further explored. It was found that the consumption of canned drinks was higher in women in which total BPA was identified in their milk samples (median (IQR) consumption of canned drinks = 2.4 servings/week (1.0–7.0) servings/week) compared to women whose milk did not show traces of BPA (median (IQR) = 1.0 servings/week (0.6–3.0) servings/week) (Kruskal-Wallis test; *p =* 0.040). 

### 3.3. Relationships between the Main and Trace Elements Content and the Microbiological and Immunological Profiles of Milk

The culture-dependent microbiological analysis indicated that the concentrations of total bacteria and total staphylococci (3.27 log_10_ CFU/mL (2.25–4.16 log_10_ CFU/mL) and 3.20 log_10_ CFU/mL (2.00–4.04 log_10_ CFU/mL), respectively) were increased among milk samples with high content of Na (≥130.09 mg/L) than in samples with low Na levels (<130.09 mg/L) (2.75 log_10_ CFU/mL (1.30–3.15 log_10_ CFU/mL) and 2.53 log_10_ CFU/mL (1.30–3.02 log_10_ CFU/mL)) (Kruskal-Wallis tests; *p* < 0.021) (Table 4). *Enterobacteriaceae* isolates were only detected in the samples with high contents of Zn (≥2.06 mg/L), but the prevalence of these microorganisms was low (9% of the samples). No strong correlations were observed between microbiological counts and the content of other main and trace elements (results not shown). Some differences were also observed in the culture-independent analysis according to the concentration of elements in milk (Table 4). The relative abundances of *Pseudomonas*, *Clostridium* and minor genera in milk samples with low Na content were about twice those found in milk samples with high Na content (Kruskal Wallis test; *p* < 0.036). A similar trend was observed in the relative abundances of *Lactobacillus*, *Bifidobacterium* and *Clostridium* according to Cu concentration in milk samples (Table 5). The relative abundances of these bacterial genera were higher among the samples with low Cu content (<0.35 mg/L) than in those samples with high Cu content (≥0.35 mg/L) (Kruskal-Wallis test; *p* < 0.046) (Table 5). Finally, higher relative abundances of *Rothia* were found among milk samples with high Cr content (≥0.32 mg/L) in comparison to samples with lower Cr content (Kruskal-Wallis test; *p =* 0.041) (Table 5). The opposite trend was observed for *Bacillus* which relative abundances doubled in the group of samples with low Cr content than in the group with more Cr (Kruskal-Wallis test; *p =* 0.022) (Table 5).

**Figure 4:**
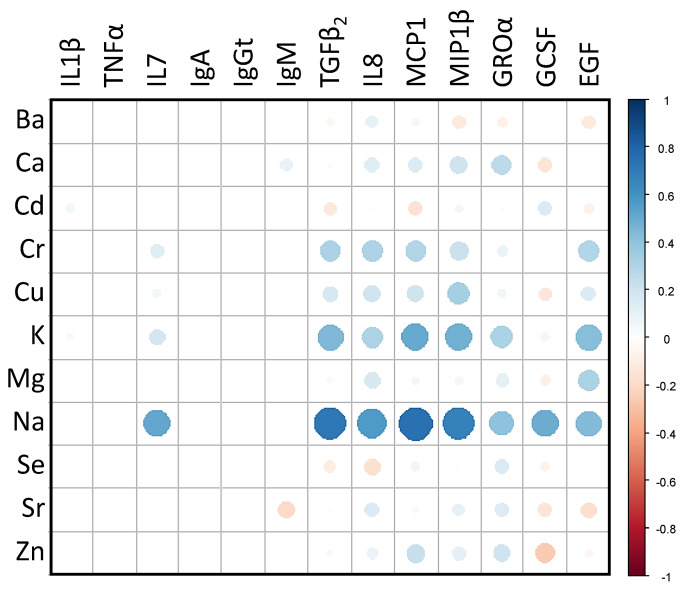
Spearman’s rank correlation matrix between the main and trace elements concentrations and the content of immunological compounds in milk samples. Blue colours show positive correlations, while reddish hues show negative correlations; dots with larger diameters show stronger correlations. The colour intensity of the dots shows the nature of the correlation: dark blue indicates a perfect positive correlation (*ρ* = 1) and dark red indicates a perfect negative correlation (*ρ* = −1).

A Spearman’s rank correlation matrix was constructed in order to find associations between the concentrations of the immune compounds and the levels of main and trace elements in milk (Figure 4). The strongest positive correlations were observed between the level of Na and IL7, TGFβ_2_, IL8, MCP1, MIP1β, GROα, GCSF and EGF concentrations (0.40 ≤ *ρ* ≤ 0.75). The correlations between K content and TGFβ_2_, IL8, MCP1, MIP1β, GROα and EGF levels were also positive, though weaker (0.31 ≤ *ρ* ≤ 0.50), than those observed for Na (Figure 4). The concentrations of most of the immunological compounds (IgM, TGFβ_2_, GROα, IL8, GCSF, MCP1, MIP1β, TNFα and EGF) were significantly higher in the group of samples with higher contents of Na (Kruskal-Wallis tests; *p* < 0.031) (Table 4). Similarly, the concentrations of IgGt and IL8 were directly correlated with the Mg content (Kruskal-Wallis tests, *p =* 0.001 and *p =* 0.004, respectively), the concentration of IgM, TGFβ_2_ and EGF with K content (Kruskal-Wallis tests, *p =* 0.011, *p =* 0.011, and *p =* 0.007, respectively), and the concentration of IgM with Cu content (Kruskal-Wallis test, *p =* 0.042) (Table 5).

### 3.4. Relationships between Free and Total BPA and TBBPA Content in Milk and the Microbial and Immunological Profiles

**Figure 5:**
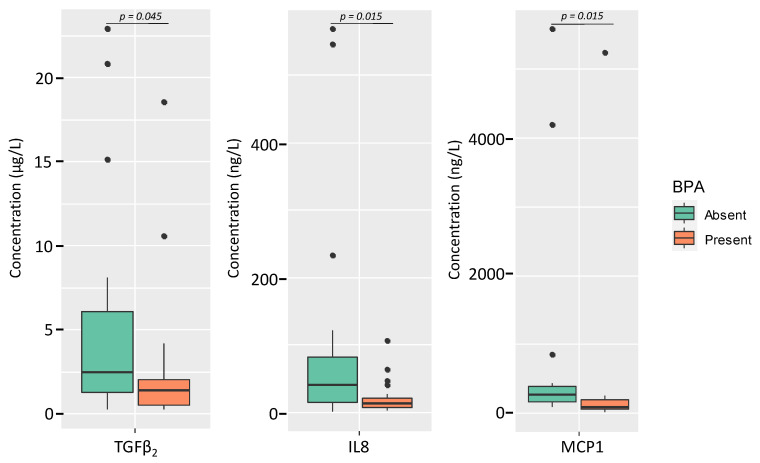
Box-plots showing the concentration of selected immunological compounds according to the presence (green) or absence (orange) of total BPA in the samples. In the box-plots, the box represents the values of the interquartile ranges, and the median is represented as a line in the box. Outliers are represented as dots. Kruskal-Wallis tests were used to evaluate differences in the concentrations of the immunological compounds between milk samples where BPA was present and those where it was absent.

There were no associations of the microbial counts and the relative abundances of bacterial phyla and species and the content of free and total BPA and TBBPA in milk (results not shown). However, regarding the immunological profile, differences were found in the levels of TGFβ_2_, IL8 and MCP1 in milk depending on the presence of BPA (Figure 5 and Supplementary Table S10). The median (IQR) concentrations of TGFβ_2_, IL8 and MCP1 were double or higher in samples where total BPA was not detected (2.48 µg/L (1.25–6.06 µg/L), 40.75 ng/L (15.20–82.97 ng/L), and 274.36 ng/L (165.18–400.04 ng/L), respectively) than in the group of samples where BPA was detected (1.41 µg/L [(0.51–2.05 µg/L), 13.70 ng/L (7.92–21.18 ng/L) and 94.65 ng/L (68.98–201.19 ng/L), respectively) (Kruskal-Wallis tests, *p =* 0.045, *p =* 0.015, and *p =* 0.015, respectively) (Figure 5 and Supplementary Table S10).

## 4. Discussion

Human milk is a very complex fluid composed of a plethora of macronutrients and micronutrients and a vast array of bioactive substances and live cells, including several subpopulations of immune cells and a site-specific microbiota [16,30,31]. Breastfeeding is unanimously recognized as the best infant feeding method in terms of health risk-benefit and independently of the mother and infant circumstances, which can include living in highly polluted environments [32,33]. Human milk may contain environmental and diet-related lipophilic chemical contaminants that accumulate in the adipose tissue (including mammary tissue) of women. These substances can be mobilized in situations in which energy and nutritional demands increase sharply, such as pregnancy or lactation. Some of these chemicals, including BPA, TBBPA and heavy metals, may act as endocrine disruptors. Therefore, their exposure above certain thresholds during foetal or early life may cause long-lasting negative effects on growth, development and health [34,35,36,37]. In this study, we elucidated whether there are potential associations between the maternal diet, the content of some major elements, trace metals, BPA and TBBPA, as well as the FAs, microbial, and immunological profiles of human milk. 

Culture-dependent analysis of the samples revealed that some of them contained high total bacterial concentrations (median total counts in the total of samples 4.77 log_10_ CFU/mL), which were mainly due to a high concentration of *S*. *epidermidis*. Increased levels of this species are usually associated with subacute or subclinical mastitis [38,39,40,41]. Although none of the women recruited in this study reported signs of mastitis, it is entirely possible that those women whose milk contained high *S*. *epidermidis* counts would be suffering from subclinical mastitis. This condition is characterized by reduced milk secretion, a high milk bacterial count, and an increase in the Na/K ratio, even in the absence of pain or other inflammation-related symptoms [42]. Sodium content and Na/K ratio have been used as biochemical markers for subclinical mastitis [43,44]. Interestingly, in our study the Na content was positively correlated with total milk bacterial counts. In addition, a positive correlation between the content of Na and some soluble immune compounds (IL8, GCSF or MIP1β) was also found. These three immune factors are known to participate in the immunological response against bacterial infection [45]. These findings reinforce the hypothesis that some of the women recruited were suffering from such a condition. Relationships between breast health status, some proinflammatory cytokines (IL1β, IL6 and IL8) and some mineral elements (P, Fe, Ca, Mg, Cu, Mn and Zn) in human milk have been previously reported [46]. Although the number of samples analysed in our studied was relatively low, our results suggest a link between breast health and the milk levels of some proinflammatory cytokines and minerals.

A potential relationship between the levels of bacteria belonging to the genera *Lactobacillus*, *Bifidobacterium*, *Clostridium* and *Bacillus* and a lower content of Cu and Cr was also noted. Long-term exposure to Cu can irritate respiratory mucous membranes, and causes headaches, nausea, and diarrhoea. In turn, high intakes can cause liver and kidney damage, and even death [47]. Cr is another hazardous heavy metal arising from industrial waste, potentially leading to pneumonia and gastrointestinal ulceration, haemorrhage and necrosis [47]. Some studies have reported the capacity of certain bacteria for binding and adsorbing metals [48,49]. It has been suggested that the sequestering of heavy metals by specific members of the human gut microbiome may greatly decrease their absorption rate, minimizing the impact of diet-related exposures even in highly polluted areas [50]. The adsorption capacities of lactobacilli and other lactic acid bacteria have been described previously [17,50,51,52,53,54]. Thus, they have been proposed for the detoxification of food and drinking water or as a probiotic for the removal of these metals once in the gut [17,53]. Subsequently, Astolfi et al. [55] tested the in vitro and in vivo binding-capacity of a multistrain probiotic product that included strains of *Lacticaseibacillus paracasei* (formerly *Lactobacillus paracasei*), *Lactiplantibacillus plantarum* (formerly *Lactobacillus plantarum*), *Lactobacillus acidophilus*, *Lactobacillus delbrueckii* subsp. *bulgaricus* and three strains of *Bifidobacterium*. They observed that the product had a good in vitro capacity to bind Cd, Hg and Pb; however, no differences in the levels of such metals were found in breast milk and infant stools when a group of women treated with the probiotic product was compared with a group taking a placebo. More in vivo studies with other lactic acid bacteria or bifidobacteria strains are necessary to understand the binding capacity of these bacteria in the human body.

In addition to lactic acid bacteria, other bacterial groups display great detoxification potential. *Bacillus* is one of the genera with the highest metal-binding activity, particularly in relation to heavy metals [49,56]. Different environmental studies have demonstrated the ability of *Bacillus licheniformis* and *Bacillus coagulans* for Cr adsorption [57]. In our study, the content of Cr was lower among the group of samples with a higher *Bacillus* concentration. 

Many studies have demonstrated the metabolic disruption produced by high or chronic BPA exposure. Unfortunately, only few addressed the impact of BPA exposure on the human microbiota, and they were mostly restricted to the use of animal models. BPA exposure in zebrafish shifted the microbiota community structure and selected BPA-resistant microbes, leading to a bacterial dysbiosis state [58,59]. Exposure to BPA can lead to gut microbiome changes in murine models [60], including a significant reduction in diversity, an increased presence of Proteobacteria, and a reduction in that of Firmicutes and Clostridia [19]. In our study, no significant differences were observed in the composition of the human milk microbiota depending on the presence of TBBPA, free BPA or total BPA in the analysed samples. This result may be due to the limited number of samples and/or to their low levels of BPA. 

Our results demonstrate the complexity of the interactions between the main parameters of the different types analysed in this work. More studies are necessary to clarify the connections among the different components of human breast milk, and to determine how these different factors affect its composition and function. 

The strengths of this study are the wide range of compounds analysed simultaneously in the same set of milk samples (main and trace elements, BPA and TBBPA, FAs, immunological compounds and microbiota), and the concomitant evaluation of the dietary habits of the women in relation to the concentration of these compounds. Studying potential relationships among these different parameters may help to understand or unveil the complex interactions that exist among the compounds that constitute human milk.

The major limitations of this study are the relatively low number of participants and the fact that all the recruited women were healthy and without a history of high or prolonged exposures to the toxic substances analysed in this work, such as heavy metals or BPA. As a consequence, the interactions observed between these compounds and the immunological and microbiological profiles were subtle. In the future, it would be interesting to have access to samples collected from women potentially exposed to environmental pollution. In addition, milk sampling may influence the fat concentration in the milk samples, and therefore the concentration of lipophilic compounds. Future studies may consider collecting more representative samples of milk and its fat content (one or more entire milkings [61]). Another limitation of the study is the lack of information on the time of day at which the samples were collected, or the time elapsed since the last feeding, as both factors strongly influence the concentration of fat in breastmilk and thus the concentration of lipophilic compounds such as BPA.

## 5. Conclusions

In this study, culture-dependent analysis of the milk samples revealed that some of them contained a high concentration of *S. epidermidis*, although none of the women recruited reported signs of mastitis that is frequently related. Our results showed a relationship between breast health and milk levels of some proinflammatory cytokines and minerals. The results of this study confirm that the microbiological composition of human milk is influenced by the dietary habits of the women. Na content was positively correlated with total milk bacterial counts and also with the following soluble immune compounds: IL8, GCSF and MIP1β. A potential relationship between the levels of bacteria belonging to the genera *Lactobacillus*, *Bifidobacterium*, *Clostridium* and *Bacillus* and a lower content of Cu and Cr was also noted in our work. The consumption of canned drinks was related with the presence of BPA in human milk. However, no significant differences were observed in the composition of the human milk microbiota depending on the presence of TBBPA, free BPA or total BPA in the analysed samples. Further studies are needed to elucidate the connections between the different components of human breast milk. In any case, breastfeeding should be always the first feeding option in early life.

## Figures and Tables

**Table 1:** Demographic characteristics of the participating women (*n* = 53).

Characteristic	*n* (%)	Mean (95% CI) or Median (IQR)
Age (years)	49	35 (34,36) ^1^
BMI (kg/m^2^)	50	24.47 (20.89–27.19)
Time of lactation (months)	43	2 (1–5)
1	16 (37)	1 (0–1)
1 to 6	19 (44)	3.00 (2.00–4.25)
>6	8 (19)	15.00 (8.25–21.75)

95% CI, 95% confidence interval; IQR, interquartile range; BMI, body mass index. Some of the anthropometric data are missing due to lack of answers in the questionnaires. Time of lactation refers to the time point when the samples were collected, and the questionnaires were conducted. ^1^ Age is expressed as mean (95% CI) and other characteristics as median (IQR).

**Table 2:** Weekly consumption (servings/food or drink item) of the different food groups among the women participating in the study (*n* = 49).

Food Group	Weekly Consumption
Dairy products	23 (16.23–31.00)
Cereals	16 (9.00–18.00)
Fish	1.5 (1.00–2.57)
Nuts	1 (0.40–3.00)
Microwavable food	0 (0.00–0.11)
Tap water	42 (28.00–56.00)
Canned drinks	2 (1.00–4.25)
Bottled water	1.5 (0.07–19.00)

All data expressed as median (IQR) servings/week of food or drink items in each food group. The dairy products group includes milk, cheese and yogurt; the cereals group includes bread, pasta and rice; and the fish group includes fresh tuna, canned tuna, salmon, swordfish and other fish. IQR, interquartile range.

**Table 3:** Frequency and concentration (expressed as µg/L) of TBBPA, free and total BPA in the milk samples depending on the dietary habits of the women participating in the study (*n* = 49).

	Cluster 1 (*n* = 19)		Cluster 2 (*n* = 22)		Cluster 3 (*n* = 8)		*p*-Value ^1^	*p*-Value ^2^
BPA (free)	5 (26)a	0.61 (0.18–0.95)	0 (0)b	-	4 (50)a	1.93 (0.19–3.78)	< 0.010	0.462
Total BPA	11 (58)	1.03 (0.60–1.63) a	9 (41)	0.32 (0.20–0.36) b	4 (50)	0.45 (0.34–4.53) a	0.553	0.048
TBBPA	2 (10)	1.60 (1.30–1.90)	0 (0)	-	1 (12)	2.30	0.192	0.221

Prevalence was expressed as the number (percentage) (n (%)) of samples in which the element was detected (relative frequency of detection). Concentration was expressed as median (IQR). ^1^ Fisher tests were used to determine a possible association between the detection of compound in milk samples and the dietary habits of the women participating in the study. ^2^ Kruskal-Wallis adjusted tests were used to determine if there were differences in the concentration of free and total BPA and TBBA between clusters. Different caption letters (a,b) mean statistical differences between clusters (*post hoc* Bonferroni adjusted test). BPA, bisphenol A; TBBPA, tetrabromobisphenol A.

**Table 4:** Frequency and concentration of microbiological counts, microbial relative abundances and immunological compounds according to the content of Na in the milk samples.

	Na < 130 mg/L		Na ≥ 130 mg/L			
	*n* (%)	Median (IQR)	*n* (%)	(Median IQR)	*p*-Value ^1^	*p*-Value ^2^
Bacterial counts (culture-dependent analysis) (*n* = 45)						
Total counts (log_10_ CFU/mL)	23 (100)	2.75 (1.30–3.15)	22 (100)	3.27 (2.25–4.16)	1.000	0.021
Total staphylococci (log_10_ CFU/mL)	22 (100)	2.53 (1.30–3.02)	21 (93)	3.20 (2.00–4.04)	0.448	0.009
Relative abundance of bacterial genera (culture-independent analysis) (*n* = 48)						
*Clostridium* (%)	23 (100)	0.07 (0.06–0.09)	25 (100)	0.04 (0.03–0.05)	1.000	0.001
*Pseudomonas* (%)	23 (100)	0.21 (0.08–0.53)	25 (100)	0.06 (0.01–0.19)	1.000	0.021
Minor genera (%)	23 (100)	13.92 (8.36–30.55)	25 (100)	6.50 (2.27–19.14)	1.000	0.036
Immunological compound (*n* = 48)						
IgM (mg/L)	23 (100)	24.26 (14.88–51.62)	25 (100)	72.79 (41.03–147.19)	1.000	< 0.001
TNFα (ng/L)	18 (78)	3.13 (1.87–4.02)	24 (96)	4.72 (2.84–10.29)	0.091	0.031
IL8 (ng/L)	23 (100)	13.70 (5.50–21.19)	25 (100)	41.30 (13.38–88.63)	1.000	0.002
GROα (µg/L)	23 (100)	0.29 (0.04–2.85)	25 (100)	3.52 (1.59–7.69)	1.000	0.002
MCP1 (ng/L)	8 (35)	94.31 (56–102.10)	20 (80)	275.24 (195.43–539.59)	0.003	0.001
MIP1b (ng/L)	18 (78)	5.24 (1.95–8.25)	25 (100)	20.97 (13.43–67.89)	0.019	< 0.001
EGF (µg/L)	23 (100)	4.96 (4.55–5.97)	25 (100)	6.08 (5.33–7.39)	1.000	0.031
GCSF (ng/L)	9 (39)	2.33 (1.67–6.54)	16 (64)	12.18 (4.55–21.86)	0.147	0.029
TGFβ_2_ (µg/L)	23 (100)	0.62 (0.31–1.76)	25 (100)	3.47 (1.61–6.23)	1.000	< 0.001

*n* (%): number (percentage) of samples in which the microorganism or compound was detected (relative frequency of detection). All data expressed as median (IQR). IQR, inter quartile range. Results from microbiological analysis are expressed as log_10_ CFU/mL. CFU, colony-forming units. Results from metataxonomic analysis are expressed as relative abundances (%). Total staphylococci include: *S. epidermidis*, *S. aureus*, *S. lugdunensis*, *S. haemolyticus*, *S. hominis* spp. *hominis* and *S. warnerii*. Minor genera include bacterial genera with a relative abundance < 0.1%. ^1^ Fisher tests were used to determine differences between the frequency of detection of microorganism or compound and the concentration detected of Na in milk samples. ^2^ Kruskal-wallis tests were used to determine differences between the concentration of each microorganism or compound and the concentration detected of Na in milk samples. EGF, epidermal growth factor; GCSF, granulocyte colony-stimulating factor; Ig, immunoglobulin; IL, interleukin; MCP1, macrophage-monocyte chemoattractant protein-1; MIP1β, macrophage inflammatory protein-1β; TGFβ_2_, transforming growth factor-β_2_; TNFα, tumor necrosis factor-α.

**Table 5:** Differences in the prevalence and concentration of microbial relative abundances obtained after metataxonomic analysis and immunological compounds according to the content of main elements in milk samples.

	Cr content					
	*n* (%)	<0.32 mg/L	*n* (%)	≥0.32 mg/L	*p*-Value ^1^	*p*-Value ^2^
*Bacillus* (%)	23 (100)	0.16 (0.10-0.52)	25 (100)	0.08 (0.05-0.16)	1.000	0.022
*Rothia* (%)	23 (100)	0.39 (0.06-2.24)	25 (100)	1.99 (0.59-4.11)	1.000	0.041
	**Cu content**					
	***n* (%)**	**<0.35 mg/L**	***n* (%)**	**≥0.35 mg/L**		
*Lactobacillus* (%)	23 (100)	0.32 (0.06-0.68)	25 (100)	0.10 (0.03-0.21)	1.000	0.046
*Clostridium* (%)	23 (100)	0.06 (0.05-0.09)	25 (100)	0.04 (0.03-0.05)	1.000	0.005
*Bifidobacterium* (%)	23 (100)	0.12 (0.05–0.71)	25 (100)	0.06 (0.01–0.10)	1.000	0.029
IgM (mg/L)	23 (100)	32.70 (17.74–69.70)	25 (100)	67.78 (34.27–138.88)	1.000	0.042
	**K content**					
	***n* (%)**	**<499.03 mg/L**	***n* (%)**	**≥499.03 mg/L**		
IgM (mg/L)	26 (100)	31.44 (17.57–69.22)	22 (100)	69.18 (40.85–130.15)	1.000	0.011
EGF (µg/L)	26 (100)	5.29 (4.38–6.06)	22 (100)	6.25 (5.26–7.65)	1.000	0.007
TGFβ_2_ (µg/L)	26 (100)	0.88 (0.32–3.10)	22 (100)	2.22 (1.64–4.06)	1.000	0.011
	**Mg content**					
	***n* (%)**	**<32.12 mg/L**	***n* (%)**	**≥32.12 mg/L**		
IgGt (mg/L)	20 (100)	49.85 (36.00–59.98)	24 (100)	90.75 (57.58–110.73)	1.000	0.001
IL8 (ng/L)	20 (100)	8.79 (4.76–26.73)	24 (100)	23.32 (14.59–70.39)	1.000	0.004

*n* (%): number (percentage) of samples in which the microorganism or compound was detected (relative frequency of detection). The relative abundances of *Bacillus*, *Bifidobacterium*, *Clostridium*, *Lactobacillus* and *Rothia* were obtained using culture-independent analysis and are expressed as percentages. All data expressed as median (IQR). ^1^ Fisher tests were used to determine differences between the frequency of detection of microorganism or compound and the concentration detected of main elements in milk samples. ^2^ Kruskal-Wallis tests were used to determine differences between the concentration of each microorganism or compound and the concentration of main elements in milk samples. EGF, epidermal growth factor; Ig, immunoglobulin; IgGt, total IgG; IQR, inter quartile range; TGFβ_2_, transforming growth factor-β_2_.

## Data Availability

The sequences of this study are available in the BioSample database of the National Center for Biotechnology Information (Bethesda, MD, USA) under the BioProject ID PRJNA715929. The rest of the data presented in this study are available on request from the corresponding author.

## Supplementary Materials

The following are available online at https://www.mdpi.com/article/10.3390/nu13124346/s1. FFQ, Food Frequency Questionnaire, Supplementary Table S1. Prevalence and bacterial counts (expressed as log_10_ CFU/mL) obtained using culture-dependent analysis of milk samples (in which bacterial growth was detected) of the women participating in the study (*n* = 48). Supplementary Table S2. Relative abundance of assigned operational taxonomic units (OTUs) at phylum and genus level (at 97% sequence similarity) in milk samples (*n* = 50). Only the most abundant OTUs present in all samples are shown. All milk samples contained OTUs assigned to the phyla and genera included in the table. Supplementary Table S3. Relative frequencies of detection and concentration of the immune factors in human milk samples (*n* = 50). Supplementary Table S4. Frequency and concentration of free and total BPA, TBBPA, major and trace elements, and individual and grouped fatty acids content in human milk samples (*n* = 49). Supplementary Table S5. Prevalence and bacterial counts (expressed as log_10_ CFU/mL) obtained using culture-dependent analysis of milk samples (where bacterial growth was detected) depending on the dietary habits of the women participating in the study (*n* = 43). Supplementary Table S6. Relative abundances of the genera detected by culture-independent analysis of milk samples depending on the dietary habits of the women participating in the study (*n* = 44). Supplementary Table S7. Prevalence and concentration of immune factors detected in human milk samples depending on the dietary habits of the women participating in the study (*n* = 45). Supplementary Table S8. Frequency and concentration (mg/L) of main and trace elements in human milk samples depending on the dietary habits of the women participating in the study (*n* = 49). Supplementary Table S9. Concentration of individual and grouped fatty acids in human milk samples depending on the dietary habits of the women participating in the study *(n* = 48). Supplementary Table S10. Prevalence and concentration of immune factors depending on the presence (*n* = 23) or absence (*n* = 21) of total BPA in human milk (*n* = 44).
