# Supplementary material for: Dietary Habits and Relationship with the Presence of Main and Trace Elements, Bisphenol A, Tetrabromobisphenol A, and the Lipid, Microbiological and Immunological Profiles of Breast Milk
Source: Nutrients. 2021 Dec 2;13(12):4346. doi: 10.3390/nu13124346 (PMC8708081; doi:10.3390/nu13124346)
Supplement: Supplementary file 1 [file nutrients-13-04346-s001.zip › nutrients-1463089-supplementary/nutrients-1463089-supplementary-1.pdf]

nutrients

MDPI

Dietary Habits and Relationship with the Presence of Main and Trace Elements, Bisphenol A, Tetrabromobisphenol A, and the Lipid, Microbiological and Immunological Profiles of Breast Milk

Irma Castro, Rebeca Arroyo, Marina Aparicio, María Ángeles Martínez, Joaquim Rovira, Susana Ares, Sara Cristina Cunha, Susana Casal, Jose Oliveira Fernandes, Marta Schuhmacher, Martí Nadal, Juan Miguel Rodríguez, Leónides Fernández

SUPPLEMENTARY INFORMATION

Supplementary Table S1. Prevalence and bacterial counts (expressed as log_{10} CFU/mL) obtained using culture-dependent analysis of milk samples (in which bacterial growth was detected) of the women participating in the study (n = 48).

Microorganism	n (%)	Bacterial counts (log_{10} CFU/mL)	Range (min - max)
Staphylococcus epidermidis	43 (90)	4.73 [3.98 - 4.99]	1.30 - 5.90
Staphylococcus aureus	12 (25)	2.68 (2.24 - 3.06)	1.30 - 4.76
Staphylococcus lugdunensis	7 (15)	3.97 [2.15 - 4.27]	2.30 - 4.41
Other staphylococci	4 (8)	2.08 (1.54 - 3.24)	1.30 - 3.80
Streptococcus parasanguinis	3 (6)	2.30 (2.02 - 3.68)	1.00 - 3.97
Streptococcus salivarius	3 (6)	2.00 (1.74 - 3.78)	1.00 - 4.08
Streptococcus mitis/oralis	2 (4)	2.66 (2.46 - 2.80)	2.08 - 2.90
Other streptococci	2 (4)	2.30 (1.95 - 3.71)	1.30 - 4.08
Corynebacterium spp.	4 (8)	3.43 (2.19 - 3.77)	1.30 - 3.90
Lactobacillus spp.	2 (4)	2.78 (2.49 - 2.95)	1.00 - 3.08
Bifidobacterium spp.	2 (4)	3.08 (2.79 - 3.25)	1.30 - 3.38
Bacillus cereus	2 (4)	3.51 (3.21 - 3.68)	1.70 - 3.81
Finegoldia magna	1 (2)	2.30	-
Gemella haemolysans	1 (2)	2.30	-
Microbacterium paraoxydans	1 (2)	3.30	-
Rothia mucilaginosa	1 (2)	3.25	-
Enterococcus faecalis	6 (12)	3.28 (2.81 - 3.62)	1.00 - 5.21
Enterobacteriaceae	5 (10)	2.37 (1.12 - 2.66)	1.60 - 2.65
Stenotrophomonas maltophilia	1 (2)	4.47	-

Prevalence was expressed as the number (percentage) [n (%)] of samples in which the microorganism was detected (relative frequency of detection). The bacterial counts were expressed either as mean [95% CI] log_{10} CFU/mL (when data were normally distributed) or median (IQR) log_{10} CFU/mL (when data were not normally distributed) (only samples where bacterial growth was detected).

Other staphylococci includes *S. haemolyticus*, *S. hominis* spp. *hominis* and *S. warnerii*. Other streptococci includes *S. gordonii* and *S. vestibularis*. *Corynebacterium* spp. includes *C. bovis* and *C. mucifaciens*. *Lactobacillus* spp. includes *L. acidophilus/gasseri* and *L. casei/paracasei*. *Enterobacteriaceae* includes *Buttiauxella agrestis, Enterobacter cloacae, Klebsiella oxytoca, Pantoea agglomerans* and *Raoutella planticale*.

CFU, colony-forming units; CI, confidence interval; IQR, interquartile range; min, minimum value; max, maximum value.

Supplementary Table S2. Relative abundance of assigned operational taxonomic units (OTUs) at phylum and genus level (at 97% sequence similarity) in milk samples (n = 50). Only the most abundant OTUs present in all samples are shown. All milk samples contained OTUs assigned to the phyla and genera included in the table.

**Phylum** Genera	**Median (IQR)**	**Range (min – max)**
Firmicutes	77.90 (48.70 - 88.86)	1.65 - 98.55
Staphylococcus	30.20 (11.08 - 63.59)	
Streptococcus	7.78 (2.14 - 31.97)	
Bacillus	0.12 (0.05 - 0.28)	
Lactobacillus	0.12 (0.04 - 0.46)	
Paenibacillus	0.12 (0.07 - 0.18)	
Macrococcus	0.09 (0.05 - 0.15)	
Enterococcus	0.06 (0.02 - 0.13)	
Clostridium	0.05 (0.04 - 0.07)	
Actinobacteria	6.76 (1.95 - 12.61)	0.16 - 44.46
Rothia	0.77 (0.08 - 2.64)	
Corynebacterium	0.52 (0.17 - 1.09)	
Propionibacterium	0.19 (0.05 - 0.63)	
Kocuria	0.08 (0.03 - 0.30)	
Bifidobacterium	0.07 (0.02 - 0.36)	
Proteobacteria	5.28 (1.77 - 25.91)	0.41 - 96.49
Pseudomonas	0.52 (0.03 - 0.28)	
Acinetobacter	0.09 (0.03 - 0.34)	
Bacteroidetes	0.30 (0.07 - 1.59)	0.02 - 13.10
Minor_phyla	0.30 (0.14 - 0.80)	0.03 - 4.88
Unclassified_phyla	0.43 (0.37 - 0.74)	0.16 - 9.34
Minor_genera	13.79 (4.45 - 25.08)	
Unclassified_genera	5.01 (3.97 - 6.41)	

Relative abundance (% of total) of OTUs. Minor phyla groups all phyla present in < 100% of the total samples with a relative abundance < 0.01%, and minor genera includes all bacterial genera present in > 15% of the samples with a relative abundance < 0.01%.

OTUs, operational taxonomic units; IQR, interquartile range; min, minimum value; max, maximum value.

Supplementary Table S3. Relative frequencies of detection and concentration of the immune factors in human milk samples (n = 50).

Immunological compound	n (%)	Median (IQR)	Range (min - max)
**Immunoglobulins**			
IgA (mg/L)	50 (100)	2796.8 (1773.5 - 4057.2)	248.40 - 13133.00
IgGt (mg/L)	50 (100)	58.22 (43.34 - 93.80)	9.10 - 274.53
IgM (mg/L)	50 (100)	48.18 (24.17 - 80.10)	6.11 - 560.04
**Innate immunity**			
IL1β (ng/L)	40 (80)	0.43 (0.16 - 1.40)	0.02 - 13.35
IL6 (ng/L)	14 (28)	24.30 (9.55 - 47.64)	0.85 - 73.45
INFγ (ng/L)	6 (12)	14.33 (6.58 - 25.06)	2.21 - 28.73
TNFα (ng/L)	44 (88)	3.53 (2.45 - 6.22)	0.26 - 39.18
**Acquired immunity**			
IL2 (ng/L)	2 (4)	13.46 (7.73 - 19.19)	2.00 - 24.92
IL4 (ng/L)	9 (18)	0.26 (0.12 - 1.26)	0.09 - 1.74
IL7 (ng/L)	39 (78)	96.22 (37.10 - 150.68)	1.84 - 315.26
IL13 (ng/L)	3 (6)	1.35 (1.29 - 2.77)	1.24 - 4.20
IL17 (ng/L)	3 (6)	9.96 (5.96 - 23.66)	1.96 - 37.37
**Chemokines**			
IL8 (ng/L)	50 (100)	17.22 (8.26 - 49.63)	1.02 - 568.46
GROα (μg/L)	50 (100)	2.97 (0.30 - 5.81)	0.02 - 13.08
MCP1 (ng/L)	29 (58)	230.38 (98.64 - 388.39)	13.95 - 5578.43
MIP1β (ng/L)	44 (88)	13.70 (4.57 - 29.70)	0.13 - 158.32
**Hematopoietic growth factors**			
EGF (μg/L)	50 (100)	5.65 (4.67 - 6.91)	3.04 - 28.18
GCSF (ng/L)	26 (52)	7.14 (2.49 - 12.99)	0.29 - 125.61
TGFβ2 (μg/L)	50 (100)	1.74 (0.60 - 4.02)	0.14 - 22.95

Prevalence was expressed as the number (percentage) [n (%)] of samples in which the immunological compound was detected (relative frequency of detection). The concentration was expressed as the median (IQR) (only samples where the immunological compound was detected).

EGF, epidermal growth factor; GCSF, granulocyte colony-stimulating factor; Ig, immunoglobulin; IgGt, total IgG; IL, interleukin; IQR, interquartile range; max, maximum value; MCP1, macrophage-monocyte chemoattractant protein-1; min, minimum value; MIP1β, macrophage inflammatory protein-1β; TGFβ2, transforming growth factor-β2; TNFα, tumor necrosis factor-α.

Supplementary Table S4. Frequency and concentration of free and total BPA, TBBPA, major and trace elements, and individual and grouped fatty acids content in human milk samples (n = 49).

Compound	n (%)	Median (IQR)
BPA (free) (μg/L)	9 (18)	0.61 (0.18-1.00)
Total BPA (μg/L)	24 (49)	0.54 (0.32-1.25)
TBBPA (μg/L)	3 (6)	2.20 (1.60-2.25)
**Elements**		
K (mg/L)	49 (100)	500.79 (439.37-580.89)
Ca (mg/L)	49 (100)	260.81 (226.52-300.35)
Na (mg/L)	49 (100)	125.22 (97.48-177.31)
Mg (mg/L)	37 (75)	33.35 (31.23-37.88)
Zn (mg/L)	27 (55)	2.09 (0.95-4.18)
Cu (mg/L)	49 (100)	0.33 (0.18-0.47)
Cr (mg/L)	49 (100)	0.31 (0.28-0.34)
Ba (mg/L)	9 (18)	0.07 (0.03-0.13)
Cd (mg/L)	7 (14)	0.06 (0.05-0.10)
Sr (mg/L)	40 (82)	0.05 (0.04-0.07)
Se (mg/L)	49 (100)	0.03 (0.01-0.06)
**Fatty acids**		
% Fat	49 (100)	3.70 (2.80-4.46)
Lauric acid (12:0) (g/L)	49 (100)	0.22 (0.14-0.34)
Myristic acid (14:0) (g/L)	49 (100)	0.21 (0.14-0.32)
Palmitoleic acid (16:1 n-7) (g/L)	49 (100)	0.06 (0.04-0.07)
Stearic acid (18:0) (g/L)	49 (100)	0.20 (0.16-0.25)
Oleic acid (18:1 n-9) (g/L)	49 (100)	1.36 (1.02-1.57)
Linoleic acid (18:2 n-6) (g/L)	49 (100)	0.43 (0.32-0.60)
α-linolenic acid (18:3 n-3) (mg/L)	49 (100)	18.63 (14.66-25.93)
ARA (20:4 n-6) (mg/L)	49 (100)	16.14 (12.12-20.69)
EPA (20:5 n-3) (mg/L)	49 (100)	1.76 (0.86-3.17)
DHA (22:6 n-3) (mg/L)	49 (100)	13.15 (7.93-17.97)
CLA (mg/L)	49 (100)	3.48 (2.64-4.69)
MUFA (g/L)	49 (100)	1.45 (1.12-1.68)
PUFA (g/L)	49 (100)	0.52 (0.42-0.74)
ω-3 PUFA (g/L)	49 (100)	0.04 (0.03-0.05)
ω-6 PUFA (g/L)	49 (100)	0.48 (0.36-0.68)
SFA (g/L)	49 (100)	1.39 (1.05-1.83)
TFA (g/L)	49 (100)	0.03 (0.02-0.03)

Prevalence was expressed as the number (percentage) [n (%)] of samples in which the compound was detected (relative frequency of detection). The concentration was expressed as the median (IQR) (only samples where the compound was detected). BPA: bisphenol A; TBBPA: tetrabromobisphenol A; ARA: arachinonic acid; EPA: eicosapentaenoic acid; DHA: docosahexaenoic acid.

Supplementary Table S5. Prevalence and bacterial counts (expressed as $\log_{10}$ CFU/mL) obtained using culture-dependent analysis of milk samples (where bacterial growth was detected) depending on the dietary habits of the women participating in the study (n = 43).

Microorganism	Cluster 1 (n = 17) n (%)	Cluster 1 (n = 17) Median (IQR)	Cluster 2 (n = 20) n (%)	Cluster 2 (n = 20) Median (IQR)	Cluster 3 (n = 6) n (%)	Cluster 3 (n = 6) Median (IQR)	*p*-value [1]	*p*-value [2]
Staphylocococcus epidermidis	15 (88)	3.07 (2.83 - 3.48)	17 (85)	3.48 (2.90 - 4.38)	6 (100)	5.06 (4.30 - 5.19)	0.839	0.135
Staphylococcus aureus	4 (31)	2.50 (1.81 - 2.91)	5 (25)	2.60 (2.30 - 2.78)	2 (33)	4.02 (3.76 - 4.18)	0.804	0.293
Total staphylococci	16 (94)	3.08 (2.73 - 3.46)	18 (90)	3.46 (2.91 - 4.38)	6 (100)	5.06 (4.30 - 5.24)	1.000	0.113
Total streptococci	4 (23)	2.20 (2.00 - 2.54)	3 (15)	2.04 (1.78 - 3.78)	0 (0)	-	0.546	0.724
Corynebacterium spp.	1 (6)	1.60	2 (10)	3.82 (3.77 - 3.86)	0 (0)	-	1.000	0.221
Enterobacteriaceae	6 (35)	2.89 (2.40 - 3.10)	2 (10)	2.48 (2.20 - 2.66)	1 (17)	3.38 (3.38 - 3.38)	0.169	0.228
Total counts	17 (100)	3.11 (2.89 - 3.81)	20 (100)	3.41 (2.73 - 4.48)	6 (100)	5.11 (4.30 - 5.25)	1.000	0.309

Prevalence was expressed as the number (percentage) [n (%)] of samples in which the microorganism was detected (relative frequency of detection). The bacterial counts are expressed as median (IQR) $\log_{10}$ CFU/mL (only samples where bacterial growth was detected). Total staphylococci includes *S. epidermidis, S. aureus, S. lugdunensis, S. haemolyticus, S. hominis* spp. *hominis* and *S. warnerii*. Total streptococcus includes *S. mitis, S. salivarius, S. parasanguinis, S. gordonii* and *S. vestibularis*. Genera *Corynebacterium* spp. includes *C. bovis* and *C. mucifaciens*. *Enterobacteriaceae* includes *Buttiauxella agrestis, Enterobacter cloacae, Klebsiella oxytoca, Pantoea agglomerans* and *Raoutella planticale*.

[1] Fisher tests were used to determine a possible association between the detection of each microorganism in milk samples and the dietary habits (cluster) of the women participating in the study.

[2] Kruskal-Wallis tests were used to determine if there were differences in bacterial concentrations in milk samples between women with different dietary habits.

IQR, interquartile range.

Supplementary Table S6. Relative abundances of the genera detected by culture-independent analysis of milk samples depending on the dietary habits of the women participating in the study (n = 44).

Genera	Cluster 1 (n = 17)	Cluster 2 (n = 20)	Cluster 3 (n = 7)	*p*-value [1]
**Firmicutes**	69.61 (47.29 - 87.57) [a]	78.26 (66.69 - 89.13) [ab]	97.09 (88.14 - 97.41) [b]	0.017
Staphylococcus	16.59 (4.17 - 38.68) [a]	44.19 (13.98 - 70.46) [ab]	89.16 (46.61 - 91.87) [b]	0.030
Streptococcus	22.27 (5.96 - 36.29)	5.75 (1.61 - 27.02)	4.55 (1.60 - 17.69)	0.254
Lactobacillus	0.32 (0.05 - 0.63)	0.16 (0.03 - 0.31)	0.06 (0.04 - 0.32)	0.519
Macrococcus	0.09 (0.04 - 0.10)	0.10 (0.05 - 0.15)	0.16 (0.12 - 0.18)	0.080
Paenibacillus	0.10 (0.06 - 0.13) [a]	0.15 (0.09 - 0.19) [ab]	0.21 (0.17 - 0.22) [b]	0.013
Bacillus	0.11 (0.09 - 0.47)	0.11 (0.05 - 0.15)	0.16 (0.06 - 0.44)	0.667
Clostridium	0.06 (0.03 - 0.08)	0.05 (0.04 - 0.08)	0.06 (0.04 - 0.08)	0.805
Enterococcus	0.07 (0.03 - 0.16)	0.03 (0.01 - 0.10)	0.07 (0.03 - 0.14)	0.209
**Proteobacteria**	8.31 (3.80 - 29.70)	7.02 (1.94 - 16.10)	1.25 (0.79 - 4.44)	0.084
Pseudomonas	0.15 (0.06 - 0.57)	0.09 (0.02 - 0.30)	0.04 (0.03 - 0.16)	0.469
**Bacteroidetes**	0.39 (0.17 - 1.60)	0.38 (0.14 - 1.97)	0.06 (0.05 - 0.43)	0.341
**Actinobacteria**	0.16 (0.09 - 4.27)	0.07 (0.03 - 0.26)	0.05 (0.02 - 0.26)	0.084
Actinobacteria	11.87 (2.75 - 13.68)	6.76 (3.42 - 9.80)	1.75 (1.15 - 6.09)	0.232
Bifidobacterium	0.09 (0.02 - 0.37)	0.09 (0.02 - 0.51)	0.02 (0.01 - 0.05)	0.250
Corynebacterium	0.89 (0.18 - 1.68)	0.47 (0.12 - 1.06)	0.28 (0.22 - 0.94)	0.665
Kocuria	0.12 (0.03 - 0.28)	0.17 (0.06 - 0.38)	0.04 (0.02 - 0.15)	0.469
Propionibacterium	0.19 (0.07 - 0.67)	0.33 (0.09 - 0.67)	0.09 (0.05 - 0.42)	0.649
Rothia	1.95 (0.44 - 4.36)	0.77 (0.10 - 2.41)	0.64 (0.19 - 1.55)	0.322
**Minor genera**	13.88 (9.58 - 19.98)	16.21 (6.06 - 34.85)	2.74 (1.34 - 16.21)	0.234
**Unclassified genera**	5.43 (4.38 - 5.61)	5.01 (3.82 - 6.39)	3.23 (3.05 - 4.11)	0.062

Relative abundance (% of total) of OTUs was expressed as median (IQR).

Minor genera groups all genera with a relative abundance below 0.01 in more than 15% of the samples.

[1] Kruskal-Wallis tests were used to determine if there were differences between the relative abundances of each operational taxonomic unit and the dietary habits of the women participating in the study. Different caption letters indicate statistical differences between clusters (*post hoc* Bonferroni adjusted test).

IQR, interquartile range; OTUs, operational taxonomic units.

Supplementary Table S7. Prevalence and concentration of immune factors detected in human milk samples depending on the dietary habits of the women participating in the study (n = 45).

Immunological compound	Cluster 1 (n = 17)		Cluster 2 (n = 21)		Cluster 3 (n = 7)		*p*-value [1]	*p*-value [2]
	n (%)	Median (IQR)	n (%)	Median (IQR)	n (%)	Median (IQR)		
**Immunoglobulins**								
IgA (mg/L)	17 (100)	2843.70 (1824.80 - 3687.40)	21 (100)	2471.60 (1646.60 - 4979.80)	7 (100)	2629.20 (1443.65 - 3499.20)	1.000	0.845
IgGt (mg/L)	17 (100)	67.16 (51.59 - 101.20)	21 (100)	48.49 (35.97 - 81.91)	7 (100)	55.98 (50.19 - 82.79)	1.000	0.221
IgM (mg/L)	17 (100)	32.70 (17.52 - 44.32)	21 (100)	70.44 (29.10 - 103.96)	7 (100)	74.57 (24.07 - 79.21)	1.000	0.066
**Innate immunity**								
IL1β (ng/L)	12 (71)	0.27 (0.08 - 0.78)	19 (91)	0.43 (0.19 - 1.04)	5 (71)	0.47 (0.14 - 0.58)	0.212	0.699
TNFα (ng/L)	15 (88)	2.94 (1.96 - 3.33) [a]	18 (86)	4.72 (3.43 - 6.42) [b]	6 (86)	2.35 (0.91 - 3.82) [ab]	0.999	0.023
**Acquired immunity**								
IL7 (ng/L)	11 (65)	85.57 (39.81 - 108.20)	18 (86)	117.37 (77.13 - 176.55)	7 (100)	48.50 (34.49 - 103.12)	0.119	0.121
**Chemokines**								
IL8 (ng/L)	17 (100)	14.74 (7.84 - 24.74)	21 (100)	17.93 (13.38 - 50.62)	7 (100)	12.68 (6.47 - 31.40)	1.000	0.403
GROα (ng/L)	17 (100)	0.40 (0.10 - 4.23)	21 (100)	3.22 (0.53 - 7.20)	7 (100)	1.66 (0.33 - 5.55)	1.000	0.545
MCP1 (ng/L)	6 (35)	99.87 (98.13 - 164.12)	14 (67)	274.36 (107.39 - 377.34)	5 (71)	105.10 (83.68 - 262.38)	0.118	0.389
MIP1β (ng/L)	14 (82)	4.96 (1.96 - 22.80)	20 (95)	13.69 (5.56 - 25.37)	6 (86)	15.82 (8.16 - 20.70)	0.359	0.411
**Hematopoietic growth factors**								
EGF (μg/L)	17 (100)	5.24 (4.40 - 5.99)	21 (100)	5.82 (5.10 - 7.28)	7 (100)	5.67 (4.47 - 6.31)	1.000	0.211
GCSF (ng/L)	7 (41)	4.86 (2.32 - 7.58)	11 (52)	6.54 (2.81 - 12.40)	4 (57)	7.36 (1.32 - 41.20)	0.720	0.837
TGFβ$_2$ (μg/L)	17 (100)	0.96 (0.31 - 2.17)	21 (100)	1.76 (0.82 - 5.44)	7 (100)	2.00 (1.72 - 2.98)	1.000	0.122

Prevalence was expressed as the number (percentage) [n(%)] of samples in which the immunological compound was detected (relative frequency of detection). The concentration was expressed as the median (IQR) (only samples where the immunological compound was detected).

[1] Fisher exact tests were used to determine a possible association between the detection of each immunological compound in milk samples and the dietary habits of the women participating in the study.

[2] Kruskal-Wallis tests were used to determine associations between the concentration of each immunological compound in milk samples and the dietary habits of the women participating in the study. Different caption letters mean statistical differences between clusters (*post hoc* Bonferroni-adjusted test).

EGF, epidermal growth factor; GCSF, granulocyte colony-stimulating factor; Ig, immunoglobulin; IL, interleukin; IQR, interquartile range; MCP1, macrophage-monocyte chemoattractant protein-1; MIP1β, macrophage inflammatory protein-1β; TGFβ$_2$, transforming growth factor-β2; TNFα, tumor necrosis factor-α.

Supplementary Table S8. Frequency and concentration (mg/L) of main and trace elements in human milk samples depending on the dietary habits of the women participating in the study (n = 49).

Element	Cluster 1 (n = 19)		Cluster 2 (n = 22)		Cluster 3 (n = 8)		*p*-value [1]	*p*-value [2]
	n (%)	Median (IQR)	n (%)	Median (IQR)	n (%)	Median (IQR)		
Ba	5 (26)	0.07 (0.07 - 0.12)	4 (18)	0.03 (0.03 - 0.05)	0 (0)	-	0.382	0.327
Ca	19 (100)	248.97 (230.45 - 285.62)	22 (100)	266.78 (227.58 - 323.00)	8 (100)	257.86 (241.25 - 300.77)	1.000	0.763
Cd	4 (21)	0.08 (0.06 - 0.11)	2 (9)	0.04 (0.04 - 0.05)	1 (12)	0.11	0.659	0.248
Cr	19 (100)	0.31 (0.27 - 0.34)	22 (100)	0.30 (0.28 - 0.33)	8 (100)	0.32 (0.30 - 0.35)	1.000	0.492
Cu	19 (100)	0.25 (0.13 - 0.42)	22 (100)	0.36 (0.26 - 0.49)	8 (100)	0.35 (0.29 - 0.42)	1.000	0.292
K	19 (100)	480.02 (438.18 - 531.45)	22 (100)	541.19 (453.13 - 597.86)	8 (100)	499.42 (446.25 - 514.34)	1.000	0.222
Mg	17 (89) [a]	34.73 (31.83 - 39.94)	18 (82) [a]	32.69 (31.04 - 37.47)	2 (25) [b]	31.87 (30.89 - 32.86)	0.003	0.574
Na	19 (100)	102.95 (83.60 - 135.25)	22 (100)	153.67 (106.59 - 235.35)	8 (100)	112.74 (82.66 - 158.53)	1.000	0.066
Se	19 (100)	0.03 (0.02 - 0.07)	22 (100)	0.03 (0.01 - 0.06)	8 (100)	0.04 (0.01 - 0.05)	1.000	0.639
Sr	17 (89)	0.05 (0.04 - 0.08)	18 (82)	0.05 (0.03 - 0.07)	5 (62)	0.07 (0.05 - 0.08)	0.228	0.251
Zn	9 (47)	1.99 (0.63 - 4.27)	14 (64)	2.42 (0.94 - 4.03)	4 (50)	2.41 (1.83 - 3.28)	0.595	0.909

Prevalence was expressed as the number (percentage) [n (%)] of samples in which the element was detected (relative frequency of detection).

[1] Fisher tests were used to determine a possible association between the detection of each major trace element in milk samples and the dietary habits of the women participating in the study. Different caption letters mean statistical differences when the *post hoc* Bonferroni adjusted test was done.

[2] Kruskal-Wallis tests were used to determine differences between the concentration detected of each major trace element and the dietary habits of the women participating in the study.

IQR, interquartile range.

Supplementary Table S9. Concentration of individual and grouped fatty acids in human milk samples depending on the dietary habits of the women participating in the study (n = 48).

Individual or grouped fatty acids	Cluster 1 (n = 18) Median (IQR)	Cluster 2 (n = 22) Median (IQR)	Cluster 3 (n = 8) Median (IQR)	*p*-value [1]
Fat (%)	3.08 (2.57 - 4.85)	3.76 (2.92 - 4.33)	4.28 (3.05 - 5.55)	0.403
Lauric acid (12:0) (g/L)	0.20 (0.09 - 0.38)	0.22 (0.17 - 0.30)	0.27 (0.18 - 0.37)	0.679
Myristic acid (14:0) (g/L)	0.16 (0.10 - 0.34)	0.21 (0.18 - 0.29)	0.26 (0.21 - 0.34)	0.582
Palmitoleic acid (16:1 n-7) (g/L)	0.05 (0.04 - 0.08)	0.06 (0.05 - 0.07)	0.06 (0.06 - 0.08)	0.405
Stearic acid (18:0) (g/L)	0.16 (0.13 - 0.25)	0.20 (0.18 - 0.23)	0.25 (0.20 - 0.27)	0.201
Oleic acid (18:1 n-9) (g/L)	1.18 (0.99 - 1.73)	1.40 (1.08 - 1.56)	1.31 (1.07 - 1.91)	0.766
Linoleic acid (18:2 n-6) (g/L)	0.42 (0.32 - 0.72)	0.44 (0.33 - 0.54)	0.49 (0.38 - 0.85)	0.692
α-Linolenic acid (18:3 n-3) (mg/L)	18.94 (16.67 - 24.65)	17.20 (13.57 - 25.33)	20.08 (15.01 - 25.85)	0.792
Arachidonic acid (20:4 n-6) (mg/L)	14.7 (11.04 - 19.76)	16.14 (12.25 - 20.67)	19.16 (14.03 - 25.26)	0.374
Eicosapentaenoic acid (20:5 n-3) (mg/L)	1.39 (0.89 - 2.66)	2.12 (0.77 - 3.07)	2.11 (1.06 - 4.72)	0.598
Docosahexaenoic acid (22:6 n-3) (mg/L)	10.67 (6.92 - 16.23)	13.25 (10 - 17.98)	14.78 (12.03 - 23.22)	0.218
CLA (mg/L)	2.74 (1.62 - 4.22)	3.72 (2.91 - 4.35)	4.58 (2.72 - 8.67)	0.098
MUFA (g/L)	1.24 (1.05 - 1.81)	1.48 (1.15 - 1.66)	1.40 (1.14 - 2.02)	0.735
PUFA (g/L)	0.51 (0.4 - 0.86)	0.56 (0.43 - 0.66)	0.60 (0.48 - 0.97)	0.686
ω-3 PUFA (g/L)	0.04 (0.03 - 0.04)	0.04 (0.03 - 0.05)	0.05 (0.03 - 0.07)	0.383
ω-6 PUFA (g/L)	0.47 (0.35 - 0.82)	0.49 (0.36 - 0.60)	0.55 (0.42 - 0.91)	0.675
SFA (g/L)	1.11 (0.83 - 2.01)	1.39 (1.18 - 1.76)	1.82 (1.15 - 2.31)	0.302
TFA (g/L)	0.02 (0.02 - 0.03)	0.03 (0.02 - 0.03)	0.03 (0.03 - 0.07)	0.097

[1] Kruskal-Wallis tests were used to determine differences between the concentration detected of individual and grouped fatty acids and the dietary habits of the women participating in the study.

IQR, interquartile range; CLA, conjugated linoleic acid; MUFA, monounsaturated fatty acids; PUFA, polyunsaturated fatty acids; SFA, saturated fatty acids; TFA, trans fatty acids.

Supplementary Table S10. Prevalence and concentration of immune factors depending on the presence (n = 23) or absence (n = 21) of total BPA in human milk (n = 44).

	**BPA absence (n = 21)**		**BPA presence (n = 23)**			
	**n (%)**	**Median (IQR)**	**n (%)**	**Median (IQR)**	***p*-value [1]**	***p*-value [2]**
TGFβ2 (μg/L)	21 (100)	2.48 (1.25 - 6.06)	23 (100)	1.41 (0.51 - 2.05)	1.000	0.045
IL8 (ng/L)	21 (100)	40.75 (15.20 - 82.97)	23 (100)	13.70 (7.92 - 21.18)	1.000	0.015
MCP1 (ng/L)	16 (76)	274.36 (165.18 - 400.04)	10 (43)	94.65 (68.98 - 201.19)	0.036	0.015

Prevalence was expressed as the number (percentage) [n (%)] of samples in which the immunological compound was detected (relative frequency of detection).

[1] Fisher tests were used to evaluate differences in the frequency of detection of each immunological compound between milk samples where BPA was present and those where it was absent.

[2] Kruskal-Wallis test were used to evaluate differences in the concentrations of the immunological compounds between milk samples where BPA was present and those where it was absent.

IL, interleukin; MCP1, macrophage-monocyte chemoattractant protein-1; TGFβ2, transforming growth factor- β2; IQR, interquartile range.
