# Supplementary material for: Dietary Habits and Relationship with the Presence of Main and Trace Elements, Bisphenol A, Tetrabromobisphenol A, and the Lipid, Microbiological and Immunological Profiles of Breast Milk
Source: Nutrients. 2021 Dec 2;13(12):4346. doi: 10.3390/nu13124346 (PMC8708081; doi:10.3390/nu13124346)
Supplement: Supplementary file 1 [file nutrients-13-04346-s001.zip › nutrients-1463089-supplementary/Supplementary material FFQ.pdf]

nutrients

MDPI

Dietary Habits and Relationship with the Presence of Main and Trace Elements, Bisphenol A, Tetrabromobisphenol A, and the Lipid, Microbiological and Immunological Profiles of Breast Milk

Irma Castro, Rebeca Arroyo, Marina Aparicio, María Ángeles Martínez, Joaquim Rovira, Susana Ares, Sara Cristina Cunha, Susana Casal, Jose Oliveira Fernandes, Marta Schuhmacher, Martí Nadal, Juan Miguel Rodríguez, Leónides Fernández

SUPPLEMENTARY INFORMATION

FOOD FREQUENCY QUESTIONNAIRE

INSTRUCTIONS

Please, fill out this questionnaire calmly. Take as much time as you consider necessary.

In this questionnaire you are asked to indicate how often you regularly consume certain foods <u>during the LACTATION period</u>.

The frequency of consumption must be specified in the boxes to the right of the list of foods in this questionnaire. For each food item in the list, you should write down <u>the number of times</u> you consume it.

- **If you consume it every day of the week, write a 7 in the "A WEEK" column. You should always add up the consumption of all the meals of the day (breakfast, lunch, snacks, dinner, other...). For example, if you drink milk every day for breakfast and sometimes for dinner: 7 + 4 = 11 times a week.**
- **If you never or rarely consume it, leave the box blank, without writing anything.**

FOOD FREQUENCY QUESTIONNAIRE

FOOD ITEM	HOW MANY TIMES	
	A WEEK	A MONTH
Milk		
Yoghurt		
Chocolate: tablet, bonbons, bars…		
Breakfast cereals ("Corn-Flakes", "Kellog's")		
Biscuits (María-type)		
Cookies, cream-filled cookies		
Muffins, sponge cake ...		
Pastry (ensaimada-type), donut, croissant...		

FOOD ITEM	HOW MANY TIMES	
	A WEEK	A WEEK
Salad: lettuce, tomato, escarole…		
Green beans, chard, spinach		
Garnish vegetables: eggplant, zucchini, mushrooms…		
Potatoes (baked, fried, boiled)		
Pulses: lentils, chickpeas, white beans…		
Rice (boiled, paella)		
	A WEEK	A MONTH
Pasta: noodles, macaroni, spaghetti…		
Soups and creams		
	A WEEK	A MONTH
Eggs		
Chicken or turkey		
Beef, pork, lamb (steak, pie…)		
Minced meat: sausage, hamburguer…		
White fish: hake, halibut…		
Blue fish: sardine, tuna, salmon …		
Seafood: mussels, shrimps, prawns, octopus, squid…		
Croquettes, pasties, pizza		
Bread (sandwich, loaf)		

FOOD ITEM	HOW MANY TIMES	
	A WEEK	A WEEK
Cured ham, cooked ham, sausages		
Fresh cheese or low-fat cheese		
Cured orsemicured cheese, cream cheese		
	A WEEK	A MONTH
Citrus fruits: orange, tangerine		
Other fruits: apple, pear, peach, apricot, ripe banana		
Canned fruit (in syrup)		
Fresh fruit juices		
Commercial fruit juices		
Nuts: peanuts, hazelnut, almonds		
Dairy desserts: custard, milk flan, cottage cheese		
Cream- or chocolate-filled pastries		
High-fat savory snacks (potato chips, Cheetos, corn chips)		
Candies or ice-cream		

	A WEEK	A MONTH
**Sugar-sweetened beverages (cola-type or other soft drinks)**		
**Low calorie beverages (soft drinks with low-calorie sweeteners…)**		
**Wine, sangría**		
**Beer**		
**Alcohol-free beer**		
**Distilled spirits (whisky, gin, brandy…)**		

	HOW MANY TIMES	
	A WEEK	A WEEK
**Do you eat in a plastic lunch or Tupperware box?**		
**Do you heat your food in a plastic lunch or Tupperware box?**		
**Do you consume food packaged in Tetrabrik or plastic containers**		
**Do you consume canned fish/seafood (tuna, sardine, cockles,**		
**Do you consume other canned food (lentils, bean stew...)**		
**Do you consume food packaged in plastic (fruit, meat or fish in**		
**Do you eat fast food?**		
